# The effect of dopaminergic treatment on whole body kinematics explored through network theory

**DOI:** 10.1038/s41598-023-50546-x

**Published:** 2024-01-22

**Authors:** Antonella Romano, Marianna Liparoti, Roberta Minino, Arianna Polverino, Lorenzo Cipriano, Anna Carotenuto, Domenico Tafuri, Giuseppe Sorrentino, Pierpaolo Sorrentino, Emahnuel Troisi Lopez

**Affiliations:** 1 Department of Medical, Motor and Wellness Sciences, University of Naples “Parthenope”, Naples, Italy; 2 Department of Philosophical, Pedagogical and Economic-Quantitative Sciences, University of Chieti-Pescara G. D’Annunzio, Chieti, Italy; 3 Institute of Diagnosis and Treatment Hermitage Capodimonte, Naples, Italy; 4 Department of Neurology, Cardarelli Hospital, Naples, Italy; 5 Institute of Applied Sciences and Intelligent Systems of National Research Council, Pozzuoli, Italy; 6 Institut de Neurosciences Des Systèmes, Inserm, INS, Aix-Marseille University, Marseille, France; 7 Department of Biomedical Sciences, University of Sassari, Sassari, Italy

**Keywords:** Neurology, Neurological disorders, Parkinson's disease, Motor control, Bone quality and biomechanics

## Abstract

Three-dimensional motion analysis represents a quantitative approach to assess spatio-temporal and kinematic changes in health and disease. However, these parameters provide only segmental information, discarding minor changes of complex whole body kinematics characterizing physiological and/or pathological conditions. We aimed to assess how levodopa intake affects the whole body, analyzing the kinematic interactions during gait in Parkinson’s disease (PD) through network theory which assess the relationships between elements of a system. To this end, we analysed gait data of 23 people with PD applying network theory to the acceleration kinematic data of 21 markers placed on participants’ body landmarks. We obtained a matrix of kinematic interactions (i.e., the kinectome) for each participant, before and after the levodopa intake, we performed a topological analysis to evaluate the large-scale interactions among body elements, and a multilinear regression analysis to verify whether the kinectome’s topology could predict the clinical variations induced by levodopa. We found that, following levodopa intake, patients with PD showed less trunk and head synchronization (*p*-head = 0.048; *p*-7th cervical vertebrae = 0.032; *p*-10th thoracic vertebrae = 0.006) and an improved upper-lower limbs synchronization (elbows right, *p* = 0.002; left, *p* = 0.005), (wrists right, *p* = 0.003; left, *p* = 0.002; knees right, *p* = 0.003; left, *p* = 0.039) proportional to the UPDRS-III scores. These results may be attributable to the reduction of rigidity, following pharmacological treatment.

## Introduction

Nowadays, the three-dimensional motion analysis, by investigating spatiotemporal, kinetic and kinematic parameters^1–3^, is considered the gold standard for fine-tuned motor assessment in health and disease (such as neurological disease), especially for what concerns gait alterations. Similarly, the development of synthetic biomechanical indices enables the analysis of more complex characteristics of gait such as the fluidity, the rhythmicity and the symmetry^4–7^.

However, the majority of these methodological approaches provide only “segmental” information, by selectively focusing only on a specific body segment such as the trunk or legs. In other words, the major constraint of these approaches is that they provide synthetic final outcomes that do not take into account the complex patterns that generated the movement itself, thus leading to a loss of information.

Therefore, an accurate characterization of movement patterns requires not only precise measurements, but also appropriate mathematical methods able to conceptualize the movement of the human body as a complex system whose components are highly interconnected to each other^8^.

Network theory is a solid, methodological framework able to describe the relationship among the elements of a complex system defining not only its properties as a whole, but also the contribution of each element within the network itself^9^. Hence, network theory could be a suitable approach to describe the whole body interactions tuned by the central nervous system in physiological conditions and in motor diseases. To this regard, the *kinectome* framework has been recently developed in order to provide a comprehensive, large-scale description of human gait kinematics through the analysis of the complex interactions among the body segments that generated the movement patterns^10^. The kinectome stems from the application of network theory to human movement^11^ and allows the investigation of the kinematic interactions occurring between anatomical segments during movement.

The kinectome has been successfully applied in Parkinson’s disease (PD)^10^. PD is now recognized as a multi-system, multi-neurotransmitter dysfunction‐related heterogeneous clinical disorder^12^. However, motor impairment, which includes reduced balance and hampered coordination, remains predominant in the clinical picture^13^.

The kinectome analysis revealed that there is a greater dysregulation in the whole-body movement pattern (i.e., higher variability) during gait with respect to non-disabled controls. Furthermore, the patients displayed greater synchronization towards the trunk, correlated to the motor clinical assessment carried out through the UPDRS-III examination. This aspect was ascribed to the typical rigidity and reduced upper limbs movement of PD^10^.

Although the pioneering studies by Hornykiewicz and Birkmayer^14^ on the use of levodopa in the treatment of PD date back to the early 1960s, levodopa remains the gold standard in therapeutic management^15^ and its efficacy in improving motor symptomatology is one of the main diagnostic criteria. For example, the assumption of levodopa results in a reduction of the variability coefficient of several spatiotemporal parameters (i.e., the stride length and the swing velocity)^16,17^. Similarly, it has been shown that, following the levodopa intake, people with Parkinson’s disease displayed an improvement of the range of motion of hip, knee, and ankle^18,19^ especially when considering the joint extension peak^20^. To this regard, Wu et al.^21^, found that, following an exogenous levodopa supplementation, the dorsiflexion and the plantarflexion of the ankle joint, were significantly improved and, more importantly this improvement was significantly correlated to the improvement of the stride length. However, while the efficacy of levodopa in relieving specific aspects of motor impairment, such as bradykinesia and rigidity^22,23^, is widely established, the drug's effects on whole body kinematics are poorly studied, resulting in a lack of information on the motor aspects that mostly impair the quality of life (i.e., balance reduction and hampered coordination).

To our knowledge, a comprehensive analysis of more complex gait features, such as motor coordination and subtle balance adjustment during gait, is still lacking^24^, similarly to a whole body network analysis assessing changes in the kinematic network following the assumption of pharmacological therapy. We hypothesize that following levodopa intake, people with PD would reduce the trunk hypersynchronization found in our previous study^10^. Furthermore, since such hypersynchronization involves the upper parts of the body, we expect to find a greater contribution of the arms to the gait.

To this aim, we used a three-dimensional motion analysis system and reflective markers, to obtain the acceleration time series of several body segments of twenty-three people with PD who were recorded before and after the intake of a subclinical dose (half of the morning dose) of levodopa. Hence, we built the kinectome by calculating the covariance between each pair of markers placed on participants’ body landmarks. Then, we performed a topological analysis to explore a possible variation of the large-scale interactions among body elements due to the assumption of the antiparkinsonian treatment. Finally, we performed a multilinear regression analysis to check whether these topological variations were related to the clinical variations assessed through the UPDRS-III.

## Materials and methods

### Participants

Twenty-three people affected by Parkison’s disease (17 males and 6 females; mean age 65.3 $\pm$ 11.58; education level 10.73 $\pm$ 3.84) (Table 1) were recruited from the Movement Disorder Unit of the Cardarelli Hospital in Naples. The PD diagnosis was defined according to the United Kingdom Parkinson’s Disease Brain Bank criteria^25^. Most of the participants did not present any lateralization of the motor symptoms, while five of them presented a slight lateralization (three on the right side, and two on the left side). Inclusion criteria were: (1) minimum age of 45 years or older; (2) Hoehn and Yahr (H&Y)^26^ score ≤ 3 while at off state (i.e., without any antiparkinsonian treatment); (3) disease duration < 10 years; (4) antiparkinsonian treatment at a stable dosage (5) absence of any neurological (except for PD) or psychiatric disorder. Exclusion criteria were: (1) Mini-Mental State Examination (MMSE) < 24^27^; (2) Frontal Assessment Battery (FAB) < 12^28^; (3) Beck Depression Inventory II (BDI-II) > 13^29^; (4) assumption of psychoactive drugs; (5) any physical or medical conditions causing walking impairment. The study protocol was approved by the local ethics committee “Azienda Ospedaliera di Rilievo Nazionale A. Cardarelli” (protocol number: 00019628) and all participants provided written informed consent in accordance with the Declaration of Helsinki.

**Table 1:** Participants characteristics.

*Parameters*	*PD*	*p-value*	
Sample size	*23*		
Age (years mean $\pm$ SD)	65.30 ($\pm$ 11.58)	n. a	
Education (years mean $\pm$ SD)	10.73 ($\pm$ 3.84)	n. a	
Gender (M/F)	17/6	n. a	
BMI (Kg/m^2^ mean $\pm$ SD)	26.1 ($\pm$ 3)	n. a	
Neuropsychological evaluation			
MMSE (mean $\pm$ SD)	28.27 ($\pm$ 1.67)	n. a	
FAB (mean $\pm$ SD)	16.96 ($\pm$ 1.96)	n. a	
BDI (mean $\pm$ SD)	6 ($\pm$ 4.15)	n. a	

Clinical evaluation	*PD off*	*PD on*	
UPDRS-III (mean $\pm$ SD)	29–17 ($\pm$ 16)	17.04 ($\pm$ 10.09)	$<$ 0.001
Disease duration (months mean $\pm$ SD)	81.78 ($\pm$ 49.92)		

MMSE: Mini Mental State Examination; FAB: Frontal Assessment Battery; BDI: Beck Depression Inventory; UPDRS-III: Unified Parkinson’s disease rating scale part three. n.a: not available.

### Stereophotogrammetric acquisition

The acquisitions took place in the Motion Analysis Laboratory (MoveNet Lab) of the University of Naples Parthenope. Gait data were obtained through a stereophotogrammetric system composed by eight infrared cameras (ProReflex Unit—Qualisys Inc., Gothenburg, Sweden) with a sampling frequency of 120 frames per second. Fifty-five passive markers were positioned on the naked skin of participants in specific anatomical landmarks according to the modified Davis protocol^30^. Through the Qualisys Track Manager (Qualisys Track Manager by Qualisys AB, Göteborg, Sweden)^31^ software we recorded the three-dimensional position of each marker (i.e., the trajectories) during the walking task. We asked the participants to walk straight at their preferred speed through a measured space (10 m)^32^. People with PD were recorded twice: during the first acquisition, they were in off state (i.e., no antiparkinsonian treatment in the last 14–16 h). The second acquisition was performed 40 min after people with PD had taken a subclinical dose (i.e., half of their usual morning intake) of levodopa (Malevodopa + Carbidopa) (on state). A subclinical dose of levodopa was administered in order to assess the kinectome sensitivity to subtle drug-induced kinematic changes. Before each acquisition each participant wes tested through UPDRS-III^33^. For both conditions (i.e., off state and on state) we recorded four trials. Each trial included two consecutive gait cycles (one for the left leg and one for the right leg).

### Data processing

The kinematic trajectories of the 55 markers were then imported into the Visual3D software (Visual 3D by C-Motion Inc., Germantown, MD)^34^. Here it was possible to build the skeletal model of the participants, interpolate short gaps in the trajectories (maximum gap 10 frames) through automated pipelines, and recognize heel strike events (through visual inspection of both trajectories and 3D video data). Each trial was then segmented starting from the heel strike of one foot, and ending in correspondence of the second heel strike of the other foot, hence obtaining a complete gait cycle per leg. Further processing and analyses we carried out on 21 markers only (see Table 2 for the anatomical correspondence of the 21 markers). We selected the more reliable markers in terms of missing data (no gaps or gaps < 10 frames), and avoided redundant information (e.g., we did not include both medial and lateral knee/elbow markers, discarding the medial one as they were more susceptible to occlusion). Data were then imported in MATLAB 2018. The time series of the marker trajectories were filtered using a lowpass 4th order butterworth filter with a 10 Hz cutoff frequency^35^. Since our aim is to evaluate whole body synchronization by the means of acceleration, this filtering approach allows us to eliminate noise that is usually present at high frequencies^36^, and that would increase with differentiation. Trajectories were then double differentiated to obtain the acceleration time series of 21 markers.

**Table 2:** Markers’ acronym and anatomical correspondence*.*

Markers’ anatomical position	Abbreviations
Head	HE
7th cervical vertebra	C7
10th thoracic vertebra	T10
Left acromion	LAC
Right acromion	RAC
Left lateral elbow	LLELB
Right lateral elbow	RLELB
Left lateral wrist	LWRB
Right lateral wrist	RWRB
Left iliac crest	LIC
Right iliac crest	RIC
Left trochanter	LGT
Right trochanter	RGT
Left lateral knee	LLK
Right lateral knee	RLK
Left lateral ankle	LLA
Right lateral ankle	RLA
Left heel	LHEEL
Right heel	RHEEL
Left 5th metatarsal	LFT5
Right 5th metatarsal	RFT5

### Kinectome analysis

**Figure 1:**
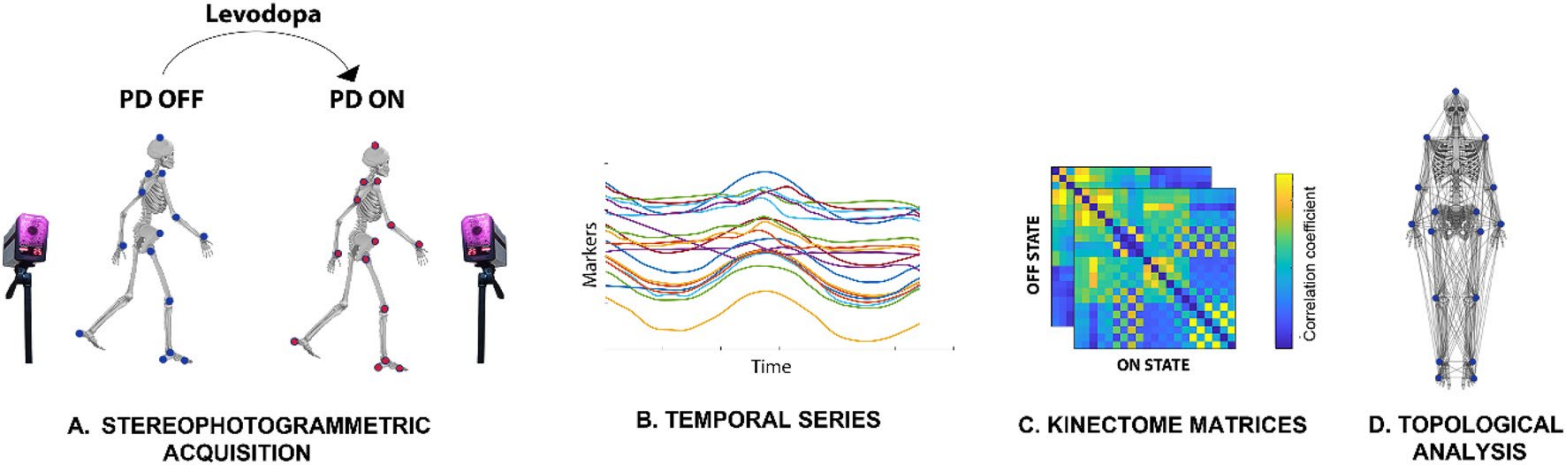
Pipeline overview. (**A**) Stereophotogrammetric acquisition. 23 people with PD were recorded through a stereophotogrammetric system before (PD-off) and after (PD-on) the assumption of levodopa. Blue and red dots represent the anatomical position of the bone marker. (**B**) Temporal series. The time series of the acceleration are obtained from the position of markers placed on participants’ body landmarks during the gait cycle. (**C**) Kinectome matrices. For each person with we obtained two kinectomes (i.e., ON state and OFF state) alongside the three axes of movement (VT, AP, ML) by computing the correlation coefficient between each pair of time series. (**D**) Topological analysis. Network representation of markers placed on participants’ body landmarks used for the topological analysis.

Here, we applied the recently developed *Kinectome* framework^10^ to provide a comprehensive description of the large-scale gait features in Parkinson’s disease and to investigate how the levodopa intake affects the large-scale movements in Parkinsonians. Overall, for each trial of each person with PD, we obtained six types of kinectomes (2 conditions × 3 axes), using the acceleration time series alongside the three axes of movement (i.e., vertical (VT), anteroposterior (AP) and mediolateral (ML). The Kinectome is a covariance matrix obtained by calculating the Pearson’s correlation coefficients between each couple of time series, among 21 markers placed on participants’ body landmarks. Hence, we identified the markers placed on participants’ body landmarks as nodes while the links (i.e., the edges) were defined by the level of synchronization between each couple of nodes (i.e., the corresponding correlation coefficient). Thereby, we obtained a symmetric matrix containing 420 edges (Fig. 1), where the elements on the diagonal are equal to 1 since they represent the correlation of a node with itself.

### Topological analysis

Through the kinectome we wanted to conceptualise the whole body as a network in which all body parts are mutually dependent. Hence, borrowing from graph theory and network analysis, we performed a topological analysis in order to assess the role of each anatomical body segment with respect to the whole body (i.e., the synchronization level of a body element with respect to the other ones). Specifically, we calculated, for each node, the nodal strength which represents the topological importance of a given node within the kinematic network^37^. The nodal strength is calculated as:

$${S}_{i}= \sum_{j=1}^{\begin{array}{c}i\ne j\\ N\end{array}}{|W}_{ij}|$$

where *i* and *j* represent two nodes in the network, *W* is the edge connecting them and *N* is the total number of the nodes of the network. Hence, the nodal strength of a given node is obtained by summing all the edges incoming to that specific node. For each type of kinectome (ML/AP/VT axes in off and on condition), the nodal strength values were averaged across the four trials.

### Multilinear regression analysis

We used the topological features obtained from the kinectome analysis in order to predict the clinical variations induced by the levodopa intake. To this end, we built a multilinear regression model in which the UPDRS-III variations (i.e., Δ-UPDRS-III) (UPDRS-III in off condition—UPDRS-III in on condition) represented the dependent variable, while age, gender, education level, disease duration (expressed in months) and the topological features of interest were the predictors. Multicollinearity was assessed through the variance inflation factor (VIF)^38,39^ which is a statistical measure used to check for highly correlated variables that would bias the results of the regression model. To validate our approach, we performed *k*-fold cross-validation, with *k* = 5^40^. Specifically, *k* iterations were performed to train our model and at each iteration the *kth* subgroup was used as a test set. The cross-validation procedure was repeated one hundred times to exclude that the result was caused by random sampling.

### Statistical analysis

Statistical analysis was carried out in Matlab (Mathworks version 2021a). The Kolmogorov Smirnov test assessed that all the data were not normally distributed^41^. A visual inspection of the distributions was performed as well. A two-sided Wilcoxon signed rank test was performed to compare the nodal strength values between the two conditions (i.e., PD-on and PD-off). The results were corrected for multiple comparisons using the False Discovery Rate (FDR) method^42^. Significance level was set at p-value after FDR correction (pFDR) < 0.05.

## Results

### Nodal strength investigation

**Figure 2:**
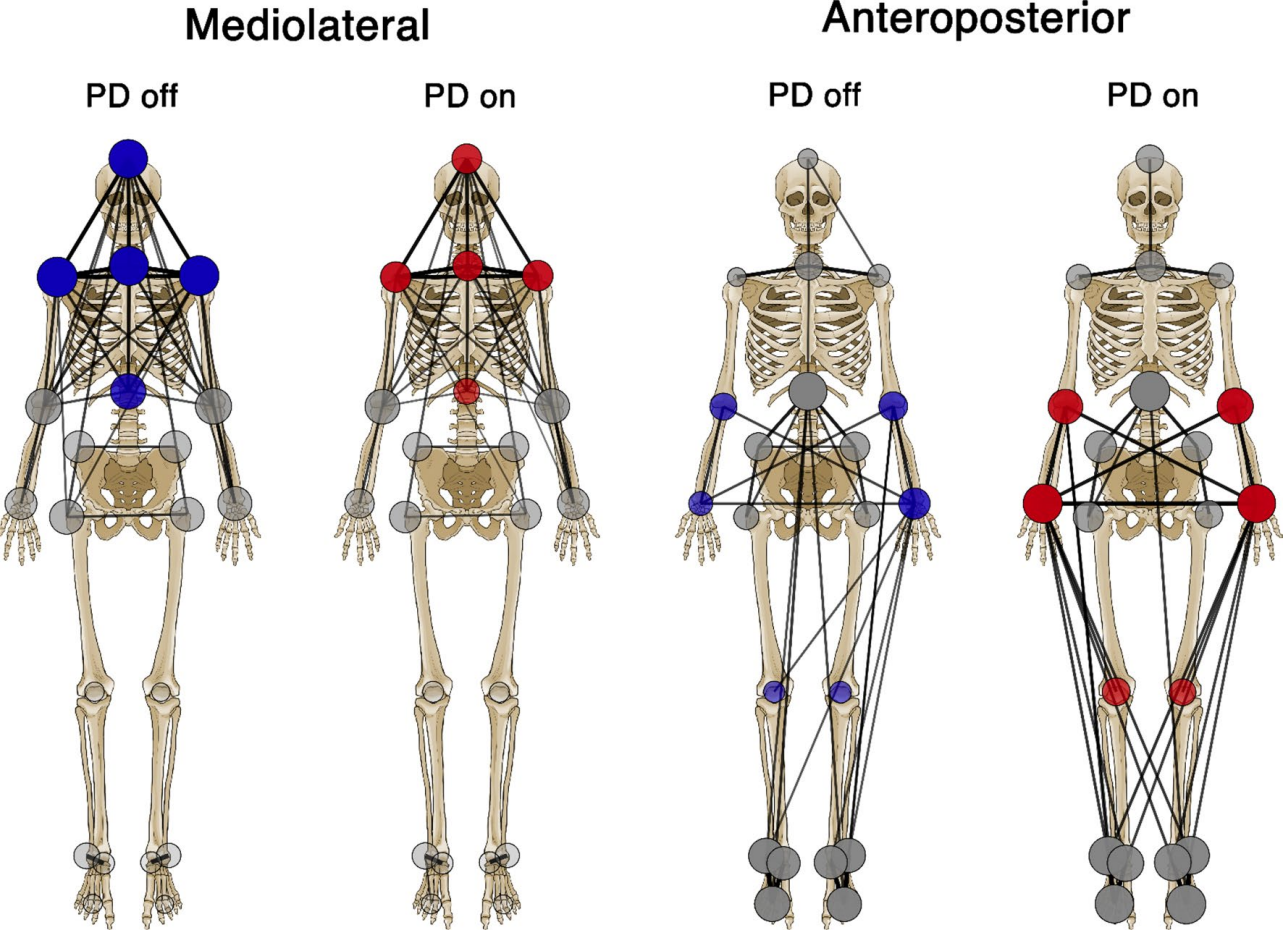
Visual representation of kinematic networks. Colored nodes represent significant differences of nodal strength between off and on conditions (i.e., without and with levodopa medicament, respectively) in people with Parkinson’s disease. Blue nodes whether synchronization was higher in off condition, red nodes whether synchronization was higher in on condition. The size and the opacity of the colored nodes depend on the nodal strength values. Gray nodes represent no significant difference. Edges are reported as black lines connecting the nodes. Please note that for visualization purpose only the 15% of the highest edges are displayed, while the statistical analysis is performed considering all the edges. The thickness and the opacity of link depend on the respective edge value.

We performed a topological analysis in order to verify whether the levodopa intake in people with PD resulted in a change of the synchronization level of a given node (i.e., a bone marker) with respect to the other body segments. We found statistical differences in ML and AP axes while we did not find any significant results on the VT axes. Concerning the ML axis, significant differences were present in the upper part of the body, showing greater synchronization in PD while in off condition. Conversely, significant results in the AP axis showed greater synchronization in on condition, and involved both upper and lower limbs. These results highlight a reduction of the ML hyper-synchronization of the trunk (i.e., rigid oscillations of the upper body), in favor of a better coordination of upper and lower limbs on the AP axis (Fig. 2).

**Figure 3:**
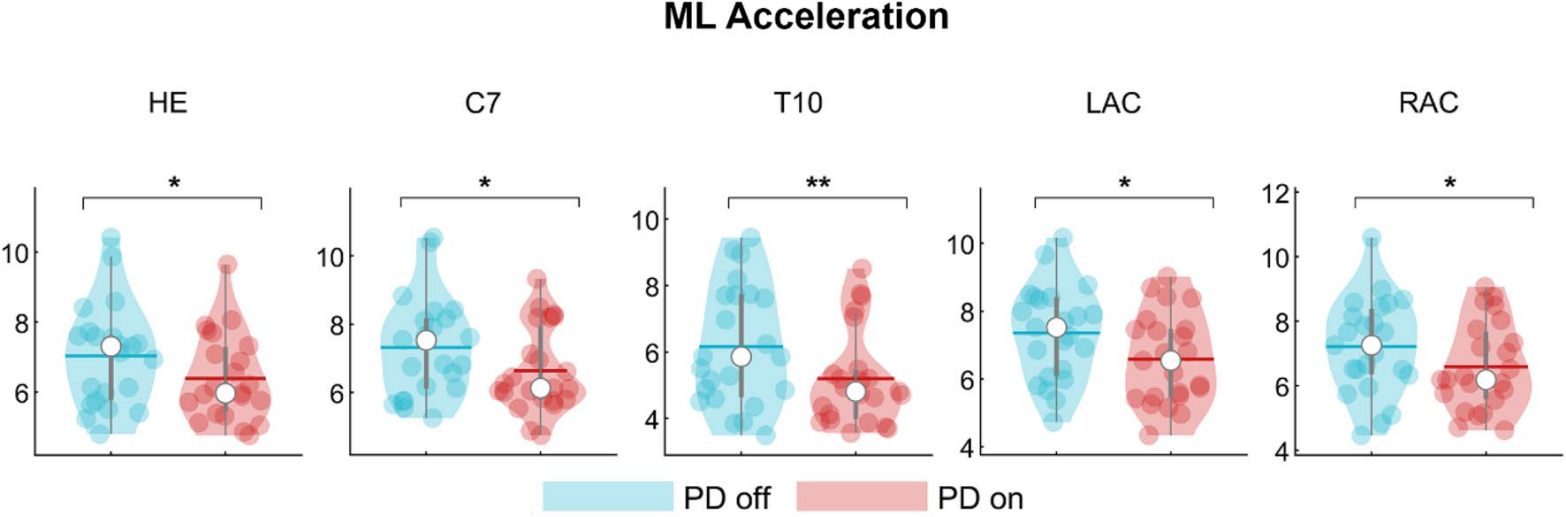
Topological comparison in mediolateral acceleration. Violin plots represent the nodal strength comparison between PD-off and PD-on conditions. People with PD during the off state showed higher values of nodal strength of the head (HE), the 7th cervical vertebra (C7), the 10th thoracic vertebra (T10) and the left (LAC) and the right (RAC) acromion with respect to the people with PD in the on state. *p < 0.05; **p < 0.01.

**Figure 4:**
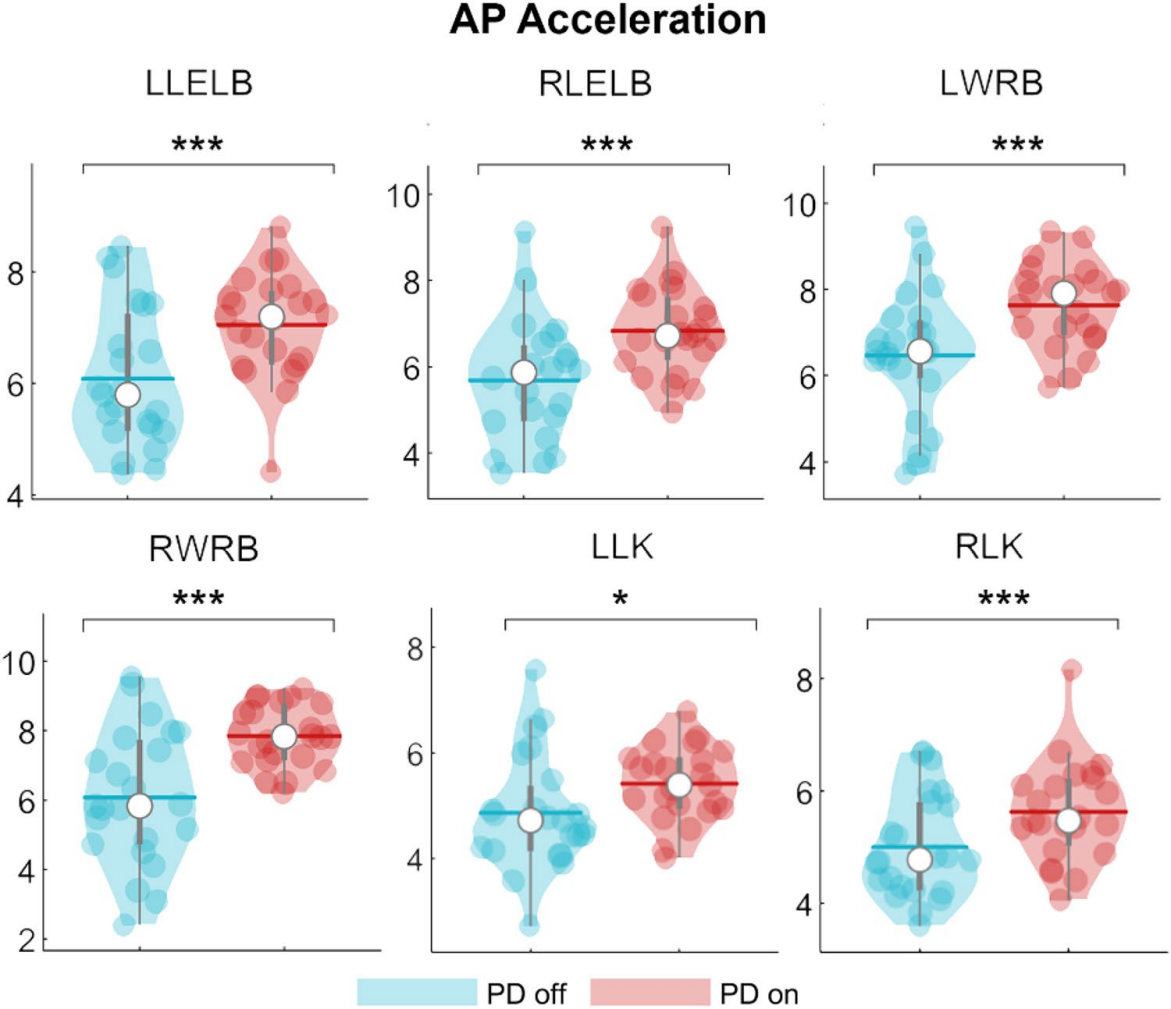
Topological comparison in antero-posterior acceleration. Violin plots represent the nodal strength comparison between PD-off and PD-on conditions Following the levodopa intake, people withPD showed higher nodal strength values of the left (LLELB) and right (RLELB) elbows, the left and right wrists (LWRB, RWRB) and left and right knees (LLK, RLK) with respect to the people with PD in the on state. *p < 0.05; ***p < 0.001.

Specifically, on the ML axis, after the levodopa intake, people with PD exhibited reduced nodal strength values with regard to the head (pFDR = 0.048), the 7th cervical vertebra (C7) (pFDR = 0.032), the 10th thoracic vertebra (T10) (pFDR = 0.006) and the right (RAC) and the left acromion (LAC) respectively (pFDR = 0.040; pFDR = 0.027) (Fig. 3). On the contrary, after levodopa intake, on the anteroposterior axis, people with PD exhibited higher nodal strength values of both the right (RLELB) and left (LLELB) elbows (pFDR = 0.002; pFDR = 0.005), the right (RWRB) and left (LWRB) wrist (pFDR = 0.003; pFDR = 0.002) and the left (LLK) and right lateral knee (RLK) (pFDR = 0.039; pFDR = 0.003) (Fig. 4). Therefore, the levodopa intake resulted in a reduction of the synchronization level of the body segments at the trunk level (i.e., lower nodal strength values) with respect to the rest of the body, and, conversely, an increase of the synchronization level (i.e., higher nodal strength values) of both the right and the left arm and the right and left knee respectively. Mean and standard deviation of nodal strength of each node are reported in the supplementary materials (Table S1 and Table S2).

### Clinical prediction

**Figure 5:**
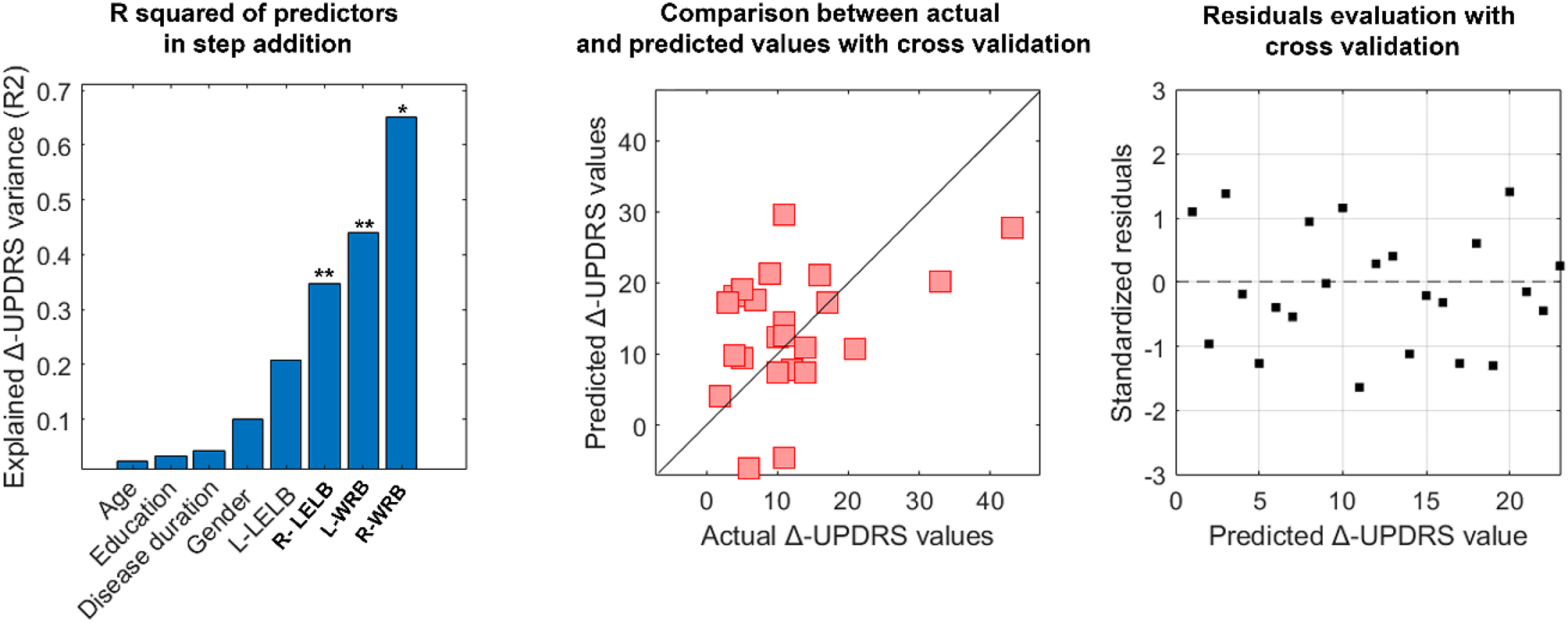
Clinical prediction. Multilinear regression analysis with *k-fold* cross validation was performed to verify the ability of the nodal strength of the upper limb to predict the clinical variation of the UPDRS-III before and after the levodopa intake (i.e., PD-off–PD -on). The left column displays the explained variance obtained by adding the predictors (age, education level, disease duration, gender and the nodal strength values of the right and left elbow and the right and left wrist). The central column displays the comparison between the predicted and the actual values of the responsive variable validated through the *k-*fold cross validation. Finally, in the right column, is displayed the distribution of residuals representing the standardisation of the difference between the actual and predicted Δ-UPDRS-III values. The significant predictors are highlighted in bold while the significant *p*-value is indicated with * (*p* < 0.05), **(*p* < 0.01).

Our previous results showed that the nodal strength of the mediolateral acceleration at T10 (ML-T10) was able to predict the clinical condition assessed through the UPDRS-III in people with PD in the off condition^10^. Hence, we aimed to test if the clinical variation (Δ-UPDRS-III) could be related to the variation of the ML-T10 nodal strength (Δ-T10). To this end, we built a multilinear regression model, validated through k-fold cross validation (k = 5) in which Δ-T10 was the predictive variabile (among other nuisance predictors such as age, education level, gender, disease duration) and the Δ-UPDRS-III was the responsive variable. The Δ-T10 did not predict the Δ-UPDRS-III (*p* = 0.332; β = 0.320; R2 = 0.17) (see supplementary materials). However, as we have previously shown, the levodopa intake resulted in a change in the motor pattern of people with PD characterised by a decrease of the synchronization at the trunk level and an increase of the synchronization level of the upper limbs in the AP axis. Hence, we wondered whether the clinical changes (Δ-UPDRS-III) could be mirrored by the AP variations of the upper limbs’ nodal strength values (i.e., Δ-RLELB; Δ-LLELB; Δ-RWRB; Δ-LWRB). Our result showed that the nodal strength variations of the right elbows and both the left and right wrist significantly contributed to the prediction of the Δ-UPDRS (RLELB *p* = 0.002, β = − 1.052; LLELB *p* = 0.009, β = − 0.768; RWRB *p* = 0.01, β = 0.888; R2 = 0.65) (Fig. 5).

## Discussion

In the present study, we used the recently developed kinectome framework to explore how the assumption of levodopa affects the large-scale kinematic interactions among body segments during gait in PD. Firstly, starting from the temporal series of the acceleration, we obtained, for each person with PD, the covariance matrices (i.e., the kinectomes) which estimated the level of synchronization between pairs of body segments (through Pearson’s correlation). In the second place, we performed a topological analysis in order to investigate the role of each human kinematic element with respect to the other ones during gait and its possible variations after the assumption of levodopa. In accordance with our hypotheses, we found that the hypersynchronization of the trunk was reduced, in favour of an improvement of the synchronization of the upper limbs. Finally, we aimed to verify whether these topological variations could be related to the clinical variation assessed through the UPDRS part III.

Our results revealed that, while in the on-state, people with PD showed a reduction of the nodal strength (i.e., lower synchronization) of the trunk (i.e., head, acromions and back) in the ML acceleration and, conversely, an increase of the nodal strength (i.,e higher synchronization) of the upper limbs (wrists and elbows) and the knees in the AP acceleration with respect to the off state. Hence, following the pharmacological treatment, people with PD exhibited a lower synchronization of the trunk with the whole body and a greater coordination between upper and lower limbs during walking. Our results are coherent with our previous findings. Indeed, in Trosi Lopez et al.^10^, we found that people with PD in off state, showed an hypersynchronization of the trunk (i.e., increased nodal strength of the 10th thoracic vertebrae). This result may be ascribed to the typical rigidity of PD. Indeed, gait in non disabled individuals is characterized by the alternation of swing and stance of the lower limbs, followed by the contralateral swing of the arms. The trunk, tied to the limbs, carries on narrower movements with respect to the limbs, with the aim to shift the weight on the stancing leg, balancing and stabilizing the whole body, improving gait smoothness^43,44^. Having high synchronization values in the trunk, indicates that several body parts move concurrently with the trunk. This reflects a less dynamic pattern of movement characterized by constrained movements, in line with the typical aspects of rigidity. However, it must be noted that we do not have information on muscular characteristics of the participant to directly assess the neurophysiological characteristics of rigidity. Here we observed that, following the assumption of levodopa, the trunk becomes more independent (i.e., less synchronized) with respect to the other body segments, suggesting that levodopa may have a role in improving the rigidity and the smoothness of gait^10,17,24,45^.

For what concerns the inter-limbic coordination, which is essential to provide dynamical stability and smoothness during gait^46^, it is disrupted in people with PD^47^. Indeed it has been shown that both ipsilateral and contralateral coordination was altered in PD and appeared to be related to a worse clinical condition (assessed through the UPDRS-III)^48^. Moreover, Winogrodzka and colleagues showed that people with PD with enhanced inter limbs coordination deficits were those in a more advanced stage of disease which displayed greater bradykinesia and rigidity in contrast with early or drug-naive people with PD who, through the manipulation of gait speed, showed a better preservation of the inter-limbic coordination^49^. Our results are in line with a previous study by Son et al.^50^ who showed that the assumption of levodopa led to an increase of the phase coordination index which was also related to a better clinical condition and a greater stability of people with PD.

Previous studies have demonstrated that levodopa treatment can reduce gait variability in PD by acting on the dopaminergic pathways^22,24,51^. For instance, Park et. al., suggested that the nigrostriatal dopaminergic modulation could play a central role in the formation of locomotor synergies (i.e., a neural organization), which are responsible for the development of movement pattern and stability^52,53^. Indeed, in another study by Carpinella et al.^54^, it has been shown that Subthalamic Nucleus Stimulation alongside the levodopa assumption led to an improvement of coordination between upper and lower limbs.

Hence, we can speculate that the improvement of motor pattern synchronization may be due to the ability of the pharmacological treatment to supply to the impairment of those brain areas involved in the synchronization and sequencing of movements such as the Basal Ganglia and/or the Supplementary Motor Area^55–58^. Further investigations are needed to deepen the actual pharmacological effects of levodopa on cortical areas involved in movement coordination.

Finally, we performed a multilinear regression analysis to check any clinical relationship between the topological variations assessed through the kinectome analysis and the UPDRS-III. The predictive models showed that, among other nuisance predictors, the nodal strength variations of the arms (i.e., left and right wrist and right elbow) was significantly able to predict the ∆UPDRS-III. We propose the idea that the increased contribution of the arms (in terms of synchronization) to the overall movement along the anteroposterior axis may be seen as a result of the reduced rigidity and bradykinesia. However, further investigation aimed to precisely assess muscular rigidity with respect to kinematic coordination should be carried on to confirm this hypothesis. A converging line of evidence assesses the role of the upper limbs in walking in both health and disease. Indeed, arms swing is essential to minimize the energy expenditure as well as to improve dynamic stability^59–62^. Intriguingly, it has been shown that upper limb movement influences the recruitment of lower limbs during rhythmic activities (e.g., walking)^60^. Please note that arm swing symmetry and coordination is disrupted in PD^63,64^. With regard to the arm-swing kinematics in PD, Navarro-López et al.^64^ carried on a review analysis concluding that there was no significant improvement of arm-swing characteristics (i.e., shoulders’ range of motion, swing amplitude, velocity, and asymmetry) following levodopa intake. However, in their study, Warmerdam et al.^65^, showed that, following the levodopa assumption, people with PD exhibited an improved arm swing, especially for what concerned the main amplitude, the peak angular velocity, coordination and sideway amplitude, suggesting, in agreement with our results, that, following pharmacological treatment, the arm swing may occur to facilitate gait pattern in people with PD. Similarly, Navarro et al.^66^ reported increased arm-swing speed and amplitude in on condition (with respect to the off condition), while Sterling et al.^67^ reported reduced asymmetry following dopaminergic treatment. Levodopa response is a key feature for the treatment of the disease and also for the diagnostic process. Having controversial results on this topic highlights the need for in-depth and multidisciplinary analysis.

This study is not without limitations. Indeed, the size of our cohort needs to be increased. Further investigation must involve more people with PD, especially women, given the gender disproportion of our cohort.

## Conclusion

In the present work we demonstrate the ability of the recently developed kinectome framework to recognize minor large-scale kinematic changes. Infact, our results revealed that, at whole body level, the levodopa intake in people with PD led to an enhanced synchronization between the upper and lower limbs which was predictive of the UPDRS-III variation. We hope that this approach may be helpful in monitoring subtle, whole body changes of PD motor characteristics during the course of the disease and with respect to the physical and pharmacological therapies. Furthermore, we highlighted the need for more evidence concerning the response to the pharmacological treatment. We believe that the kinectome approach may be of help in future experimental settings aimed to evaluate the kinematic coordinative mechanisms in relation to other symptomatic aspects of Parkinson's and its response to levodopa.

### Supplementary Information

Supplementary Information 1.

## Data Availability

The datasets generated and/or analysed during the current study are not publicly available due to the clinical nature of the cohort under study but are available from the corresponding author on reasonable request.
