# Supplementary material for: The effect of dopaminergic treatment on whole body kinematics explored through network theory
Source: Sci Rep. 2024 Jan 22;14:1913. doi: 10.1038/s41598-023-50546-x (PMC10803322; doi:10.1038/s41598-023-50546-x)
Supplement: Supplementary file 1 — Supplementary Information 1. [file 41598_2023_50546_MOESM1_ESM.docx]

**Supplementary materials**

**
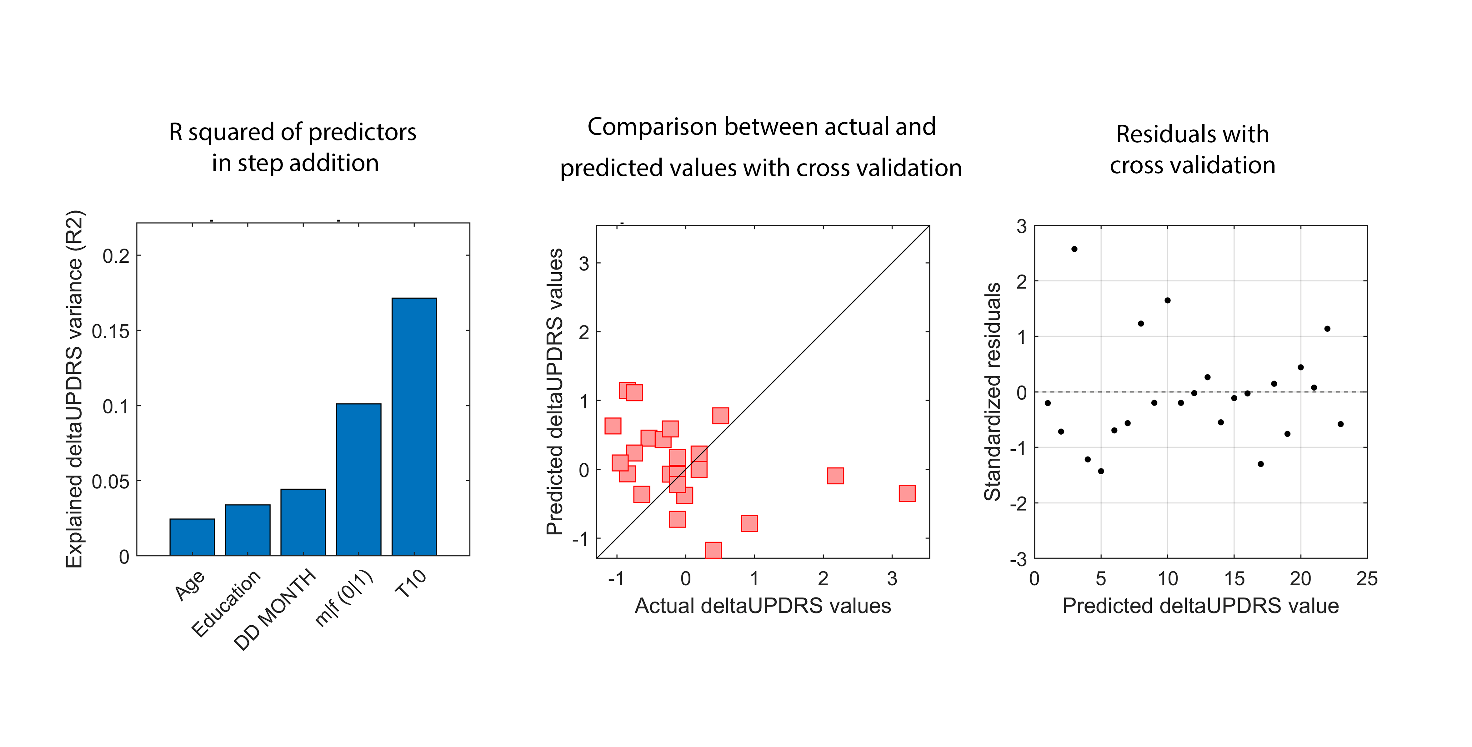
**

**Clinical prediction.** Multilinear regression analysis with *k-fold* cross validation was performed to verify the ability of the nodal strength of T10 to predict the clinical variation of the UPDRS-III before and after the levodopa intake (i.e., PD-off - PD -on). The left column displays the explained variance obtained by adding the predictors (age, education level, disease duration, gender and nodal strength of T10). The central column displays the comparison between the predicted and the actual values of the responsive variable validated through the *k-*fold cross validation. Finally, in the right column, is displayed the distribution of residuals representing the standardisation of the difference between the actual and predicted Δ-UPDRS-III values. As displayed in the predictive models, none of the predictors, including the delta value of the T10 nodal strength (i.e., T10 off – T10 on), were able to predict the clinical variations assessed by the UPDRS-III.

**Table S1: Mean and standard deviation (SD) on mediolateral acceleration of 21 nodes.**

| **NODES** | **Off condition**  **(mean** $\boldsymbol{\pm}$ **SD)** | **On condition**  **(mean** $\boldsymbol{\pm}$ **SD)** | **p value** |
| --- | --- | --- | --- |
| Head (HE) | 5.57 (± 1.54) | 5.84 (± 1.46) | n.s |
| 7^th^  cervical vertebra (C7) | 6.24 (± 1.46) | 6.17 (± 1.67) | n.s |
| 10^th^ thoracic vertebra (T10) | 7.54 (± 1.27) | 7.88 (± 1.10) | n.s |
| Left acromion (LAC) | 5.24 (± 1.44) | 5.37 (±1.31) | n.s |
| Right acromion (RAC) | 5.45 (± 1.35) | 5.84 (±1.38) | n.s |
| Left lateral elbow (LLELB) | 6.08 (± 1.25) | 7.041 (±0.92) | <0.01 |
| Right lateral elbow (RLELB) | 5.68 (± 1.38) | 6.83 (± 0.98) | <0.01 |
| Left lateral wrist (LWRB) | 6.47 (± 1.47) | 7.63 (± 0.94) | <0.01 |
| Right lateral wrist (RWRB) | 6.07 (± 1.94) | 7.84 (± 0.88) | <0.01 |
| Left iliac crest (LIC) | 6.16 (± 1.23) | 5.97 (± 0.97) | n.s |
| Right iliac crest (RIC) | 6.06 (± 1.29) | 6.20 (± 1.38) | n.s |
| Left trochanter (LGT) | 5.42 (± 1.07) | 5.39 (± 0.80) | n.s |
| Right trochanter (RGT) | 5.36 (± 1.16) | 5.77 (± 1.13) | n.s |
| Left lateral knee (LLK) | 4.86 (± 1.09) | 5.41 (± 0.68) | <0.01 |
| Right lateral knee (RLK) | 5.00 (± 0.93) | 5.63 (± 0.87) | <0.01 |
| Left lateral ankle (LLA) | 6.75 (± 0.86) | 7.14 (± 0.89) | n.s |
| Right lateral ankle (RLA) | 6.67 (± 1.04) | 7.23 (± 0.86) | n.s |
| Left heel (LHEEL) | 6.54 (± 0.74) | 6.96 (± 0.45) | n.s |
| Right heel (RHEEL) | 6.30 (± 0.95) | 6.80 (± 0.77) | n.s |
| Left 5^th^ metatarsal (LFT5) | 6.70 (± 0.96) | 7.35 (± 0.81) | n.s |
| Right 5^th^ metatarsal (RFT5) | 6.62 (± 1.09) | 7.14 (± 0.92) | n.s |

**Table S2: Mean and standard deviation (SD) on anteroposterior acceleration of 21 nodes**

| **NODES** | **Off condition**  **(mean** $\boldsymbol{\pm}$ **SD)** | **On condition**  **(mean** $\boldsymbol{\pm}$ **SD)** | **p value** |
| --- | --- | --- | --- |
| Head (HE) | 7.03 ($\pm$ 1.43) | 6.39 ($\pm$ 1.22) | < 0.05 |
| 7^th^  cervical vertebra (C7) | 7.31 ($\pm$ 1.44) | 6.63 ($\pm$1.24) | < 0.05 |
| 10^th^ thoracic vertebra (T10) | 6.16 ($\pm$1.82) | 5.19 ($\pm$ 1.45) | <0.01 |
| Left acromion (LAC) | 7.35 ($\pm$ 1.38) | 6.58 ($\pm$1.32) | <0.05 |
| Right acromion (RAC) | 7.21 ($\pm$ 1.47) | 6.59 ($\pm$ 1.34) | <0.05 |
| Left lateral elbow (LLELB) | 6.26 ($\pm$ 1.62) | 5.77 ($\pm$ 1.09) | n.s |
| Right lateral elbow (RLELB) | 6.17 ($\pm$ 1.32) | 5.50 ($\pm$ 1.08) | n.s |
| Left lateral wrist (LWRB) | 5.65 ($\pm$ 1.64) | 5.13 ($\pm$ 1.23) | n.s |
| Right lateral wrist (RWRB) | 5.24 ($\pm$ 1.55) | 4.56 ($\pm$ 1.08) | n.s |
| Left iliac crest (LIC) | 5.38 ($\pm$ 1.35) | 4.78 ($\pm$ 1.44) | n.s |
| Right iliac crest (RIC) | 5.31 ($\pm$ 1.41) | 4.65 ($\pm$ 1.25) | n.s |
| Left trochanter (LGT) | 5.75 ($\pm$ 1.50) | 5.07 ($\pm$ 1.42) | n.s |
| Right trochanter (RGT) | 5.66 ($\pm$ 1.46) | 5.06 ($\pm$1.26) | n.s |
| Left lateral knee (LLK) | 3.19 ($\pm$ 0.75) | 3.35 ($\pm$ 0.82) | n.s |
| Right lateral knee (RLK) | 3.38 ($\pm$ 0.80) | 3.41 ($\pm$ 0.78) | n.s |
| Left lateral ankle (LLA) | 3.67 ($\pm$ 0.68) | 3.71 ($\pm$ 0.86) | n.s |
| Right lateral ankle (RLA) | 3.99 ($\pm$ 0.72) | 3.97 ($\pm$ 0.73) | n.s |
| Left heel (LHEEL) | 3.32 ($\pm$ 0.69) | 3.44 ($\pm$ 0.70) | n.s |
| Right heel (RHEEL) | 3.40 ($\pm$ 0.67) | 3.42 ($\pm$ 0.72) | n.s |
| Left 5^th^ metatarsal (LFT5) | 2.79 ($\pm$ 0.66) | 2.85 ($\pm$ 0. 79) | n.s |
| Right 5^th^ metatarsal (RFT5) | 2.63 ($\pm$ 0.63) | 2.91 ($\pm$ 0.65) | n.s |
